# Reduction in IGF1 mRNA in the Human Subependymal Zone During Aging

**DOI:** 10.14336/AD.2018.0317

**Published:** 2019-02-01

**Authors:** Christin Weissleder, Guy Barry, Samantha J. Fung, Matthew W. Wong, Kay L. Double, Maree J. Webster, Cynthia Shannon Weickert

**Affiliations:** ^1^Schizophrenia Research Laboratory, Neuroscience Research Australia, Randwick, NSW, Australia.; ^2^School of Psychiatry, Faculty of Medicine, University of New South Wales, Sydney, NSW, Australia.; ^3^QIMR Berghofer Medical Research Institute, Herston, QLD, Australia.; ^4^School of Medical Sciences, University of New South Wales, Sydney, NSW, Australia.; ^5^Discipline of Biomedical Science and Brain and Mind Centre, Sydney Medical School, University of Sydney, Australia.; ^6^Laboratory of Brain Research, Stanley Medical Research Institute, Maryland, USA

**Keywords:** neurogenesis, subventricular zone, doublecortin, Ki67, IGF binding proteins

## Abstract

The cell proliferation marker, Ki67 and the immature neuron marker, doublecortin are both expressed in the major human neurogenic niche, the subependymal zone (SEZ), but expression progressively decreases across the adult lifespan (PMID: 27932973). In contrast, transcript levels of several mitogens (transforming growth factor α, epidermal growth factor and fibroblast growth factor 2) do not decline with age in the human SEZ, suggesting that other growth factors may contribute to the reduced neurogenic potential. While insulin like growth factor 1 (IGF1) regulates neurogenesis throughout aging in the mouse brain, the extent to which IGF1 and IGF family members change with age and relate to adult neurogenesis markers in the human SEZ has not yet been determined. We used quantitative polymerase chain reaction to examine gene expression of seven IGF family members [IGF1, IGF1 receptor, insulin receptor and high-affinity IGF binding proteins (IGFBPs) 2, 3, 4 and 5] in the human SEZ across the adult lifespan (n=50, 21-103 years). We found that only IGF1 expression significantly decreased with increasing age. IGFBP2 and IGFBP4 expression positively correlated with Ki67 mRNA. IGF1 expression positively correlated with doublecortin mRNA, whereas IGFBP2 expression negatively correlated with doublecortin mRNA. Our results suggest IGF family members are local regulators of neurogenesis and indicate that the age-related reduction in IGF1 mRNA may limit new neuron production by restricting neuronal differentiation in the human SEZ.

The human brain retains the ability to generate new neurons throughout postnatal life with the largest reservoir of newly-born cells in the subependymal zone (SEZ, also subventricular zone) [1]. Neurogenesis in the human SEZ after infancy has been called into question [2, 3] despite evidence supporting the existence of cells in different stages of neurogenesis throughout adulthood from both our laboratory and other groups [4-8]. Although the functional significance of neurogenesis in the human SEZ remains to be established, the putative integration of newly-generated interneurons into subcortical and cortical brain regions may contribute to synaptic plasticity and cognitive flexibility [9-12]. Cell culture experiments have advanced our understanding of the molecular control of neurogenesis by identifying growth factors as key regulators of proliferation and cell fate decision. However, the extent to which growth factors and their receptors are altered with age in the human SEZ in parallel with the decline in cell proliferation and neuronal differentiation markers is only partially characterized [5, 7, 13]. Transcript levels of transforming growth factor α, epidermal growth factor, fibroblast growth factor 2 and cognate receptors do not decrease during aging [7, 14] and point to the involvement of other mitogens such as insulin like growth factor (IGF) 1 and 2.

IGF1 and 2 transcripts decrease in most regions of the rodent nervous system during postnatal life but levels remain elevated in adult neurogenic regions [15, 16]. IGF1 is expressed by neurons [17], astrocytes [18] and oligodendrocytes [19], whereas IGF2 is produced by the leptomeninges and choroidal epithelial cells [20]. IGF1 signals predominantly through the IGF1 receptor (IGF1R) but can also bind with low affinity to the insulin receptor (INSR). IGF1R expression predominates in neuronal precursor cells, whereas INSR is abundantly expressed by neural stem cells in the adult SEZ [21]. IGF1 bioavailability is regulated by high- and low-affinity IGF binding proteins (IGFBPs), which are expressed by endothelial cells, neurons and glia [22, 23]. IGFBP3 and IGFBP4 modulate neural precursor proliferation, differentiation and survival [24, 25].

IGF1 and IGFBPs are expressed in the peripheral blood and cerebrospinal fluid and show distinct age-related alterations [26-29]. The entrance of peripheral IGF1 and insulin into the brain parenchyma is controlled by IGF1R, INSR, IGFBPs and low-density lipoprotein receptor-related proteins [30], and influences neurogenesis and cognition [31-33]. Werry and colleagues did not detect a change in IGF1 protein levels in the SEZ from adulthood into aging [14]; however, the amount of IGF1 available to signal could be impacted by age-related alterations in IGFBPs without a change in IGF1 itself. Since brain IGF1 protein levels can also be derived from the peripheral blood and cerebrospinal fluid, we hypothesized that local production of IGF1 and IGF family members would be reduced in the human SEZ and would correlate with expression of cell proliferation and neuronal differentiation markers.

## MATERIALS AND METHODS

### Human post-mortem brain samples

Tissue from the anterior caudate of 50 healthy individuals was obtained from the Stanley Medical Research Institute and New South Wales Brain Tissue Resource Centre (Sydney, Australia; HREC 12435, HC16442). Cases had no known history of psychiatric symptoms or substance abuse and showed no significant neuropathology on post-mortem examination. The brain cohort consisted of 9 females and 41 males, with an average age of 52 years (±16.76, range 21-103 years), average pH of 6.59 (±0.25, range 5.95-7.03) and average post-mortem interval (PMI) of 29 hours (±10.75, range 9-58 hours). Demographic details of each individual have been described previously [7].

### Processing of brain tissue

Fresh-frozen caudate tissue was sectioned on a Leica CM3050 S cryostat, taking 20 *x* 60 µm sections interspersed with 10 *x* 14 µm sections. SEZ tissue was dissected from the caudate nucleus while frozen over dry ice from 60 µm thick sections, ~2 mm deep to the surface of the lateral ventricle. For each case, tissue was dissected from 3 sets of 3-4 adjacent 60 µm sections spaced ~1340 µm to give 10 sections per case (~40 mg tissue total).

### RNA extraction and cDNA synthesis

Total RNA was extracted for all cases using Trizol (Life Technologies). RNA quality and concentration were assessed with Agilent Technologies 2100 Bioanalyzer and Nanodrop ND-1000 spectrophotometer. The average RNA integrity number (RIN) was 7. cDNA was synthesized from 3 µg total RNA per case using SuperScript® First-Strand Synthesis kit and random hexamers (Life Technologies).

### Assessment of mRNA expression of IGF family members using quantitative reverse transcription polymerase chain reaction

mRNA levels were measured by TaqMan Gene Expression Assays (Applied Biosystems; IGF1, Hs01547656_m1; IGF1R, Hs00609566_m1; IGFBP2, Hs01040719_m1; IGFBP3, Hs00181211_m1; IGFBP4, Hs01057900_m1; IGFBP5, Hs00181213_m1; INSR, Hs00961557_m1) using an ABI Prism 7900HT fast real-time PCR system and a 384-well format. All measurements from each subject were performed in duplicate and relative quantities were determined from a seven-point standard curve of pooled cDNA. The no template controls did not produce a signal for any mRNA examined. Expression of two housekeeping genes, TATA-box binding protein (Hs00427620_m1) and ubiquitin C (Hs00824723_m1), was used to calculate the normalizing factor for gene expression (geometric mean), and neither of these mRNAs nor the geometric mean correlated significantly with age (all p>0.05, data not shown). Quantitative reverse transcription polymerase chain reaction data were captured with sequence detector software (SDS version 2.4, Applied Biosystems). SDS software plotted real-time fluorescence intensity and the threshold was set within the linear phase of the amplification profiles.

### Statistics

Statistical analyses were performed using IBM SPSS Statistics Version 24 and GraphPad Prism Version 7.0 B. Results were considered as significant at an α level of p<0.05. Population outliers were defined as points lying outside of a 95% prediction interval from the linear regression line (1-3 individuals per target gene). Data were tested for normality using the Shapiro-Wilk test. Pearson’s product-moment correlations were used to investigate the relationships of brain cohort characteristics (age, pH, PMI and RIN) to each other and target gene expression. Pearson’s product-moment or semi-partial correlations were used to analyze age-related changes in target gene expression and their relationships to markers of cell proliferation and neuronal differentiation. When semi-partial correlations were performed, the semi-partial correlation coefficient sr is reported. Independent *t*-tests or analysis of variance co-varying for brain cohort characteristics were used as appropriate to detect sex-related differences in gene expression, and sex did not show a significant effect on gene expression (p>0.05).

## RESULTS

### Expression of IGF family members in the human SEZ from young adulthood into aging

**Figure 1.**
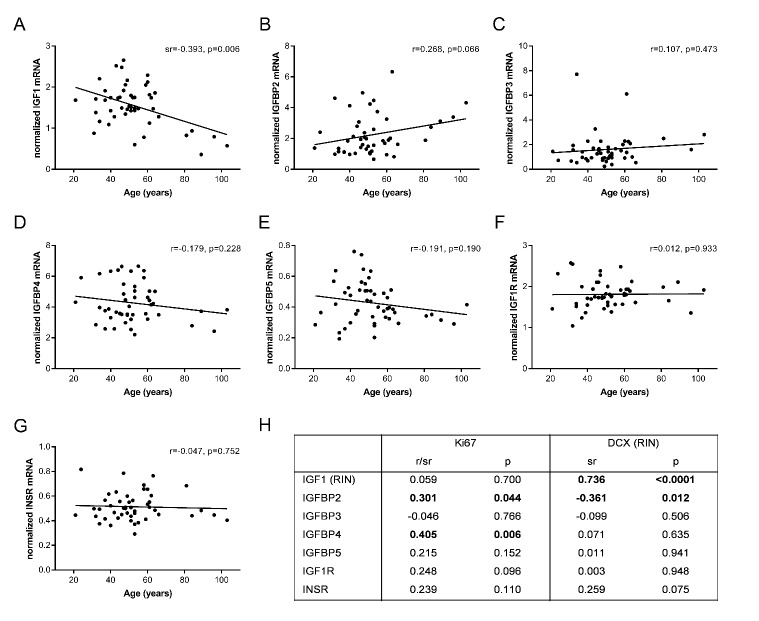
**Gene expression of IGF family members and their relationships to neurogenesis markers in the human SEZ from young adulthood into aging.** IGF1 mRNA significantly decreased in the aging SEZ (A). IGFBP2 mRNA showed a trend increase with age (B), while expression of IGFBPs 3-5, IGF1R and INSR remained stable throughout adulthood (C-G). Pearson’s product-moment and semi-partial correlations demonstrated different relationships between IGF family member expression and cell proliferation (Ki67) and immature neuron markers (DCX). Confounding brain cohort characteristics (in brackets) were considered as covariates in semi-partial correlation analyses (H). RIN, RNA integrity number; sr, semi-partial correlation coefficient. Bold type = p≤0.05.

Gene expression of seven out of ten IGF family members was reliably detected by quantitative polymerase chain reaction in the adult human SEZ from 21-103 years. Transcript levels of IGFBP1, IGF2 and IGF2 receptor were below the level of detection. IGF1 mRNA decreased significantly with age (sr=-0.39, p=0.006; Fig. 1A). IGFBP2 mRNA showed a trend towards an increase with age (r=0.26, p=0.06; Fig. 1B), whereas IGFBP3, IGFBP4 and IGFBP5 mRNAs did not change across the adult lifespan (all p≥0.19; Fig. 1C-E). Transcript levels of IGFBPs did not correlate with IGF1 mRNA (all p≥0.09, data not shown). IGF1R and INSR mRNAs remained stable throughout adulthood (all p≥0.75; Figs. 1F, G).

**Table 1:** Pearson’s product-moment correlations between gene expression of IGF family members and brain cohort characteristics in the human SEZ.

	pH		PMI		RIN	
	r	p	r	p	r	p
IGF1	**0.518**	<**0.0001**	-0.072	0.628	**0.332**	**0.021**
IGFBP2	-**0.361**	**0.012**	-0.021	0.890	-0.137	0.354
IGFBP3	-0.206	0.165	-0.229	0.122	0.185	0.214
IGFBP4	-0.180	0.226	-0.031	0.837	-0.218	0.141
IGFBP5	0.213	0.141	0.120	0.412	-0.085	0.561
IGF1R	-**0.325**	**0.023**	-0.198	0.172	-0.177	0.224
INSR	-0.046	0.756	0.263	0.071	-0.014	0.923

PMI, post-mortem interval; r, Pearson’s product-moment correlation coefficient; RIN, RNA integrity number. Bold type = p≤0.05.

### Relationships of IGF family member transcripts to decreased expression of adult neurogenesis markers

We analyzed the relationships between expression of IGF family members and expression of the cell proliferation marker Ki67 and of the immature neuron marker doublecortin (DCX) (Fig. 1H), which we have previously shown to progressively decline with age in the SEZ [7]. Ki67 mRNA positively correlated with IGFBP2 (r=0.30, p=0.04) and IGFBP4 mRNAs (r=0.40, p=0.006). IGF1 mRNA was strongly positively correlated with DCX mRNA (sr=0.73, p<0.0001), whereas IGFBP2 mRNA showed a negative relationship with DCX mRNA (sr= -0.36, p=0.01).

### Correlations between brain cohort characteristics and target gene expression

Detailed statistical data for the relationships between brain cohort characteristics and target gene expression are presented in Table 1. Brain pH correlated with IGF1 (r=0.51, p<0.0001), IGF1R (r=-0.32, p=0.02) and IGFBP2 mRNAs (r=-0.36, p=0.01). RIN positively correlated with IGF1 mRNA (r=0.33, p=0.02). No other significant relationships were detected between brain cohort characteristics and target gene expression. The relationships between age and other brain cohort characteristics (pH, PMI and RIN) have been described previously [7]. Briefly, age negatively correlated with pH (r=-0.43, p=0.002). No other significant relationships were detected between age, PMI, pH and RIN (all p>0.05, data not shown). Brain pH was excluded as a covariate during statistical analyses since aging is commonly associated with brain acidosis and thus dependent on the variable of interest.

## DISCUSSION

This study provides the first molecular evidence for an age-related reduction in local IGF1 expression in the human SEZ from adulthood into aging and lends translational support for the fundamental work in rodents. Our results suggest that subependymal cells remain responsive to IGF1 as indicated by stable expression of cognate receptors IGF1R and INSR. We found that IGFBP2 and IGFBP4 may be important in the regulation of cell proliferation, while IGF1 may promote neuronal differentiation. This study only allows evaluation of transcriptional alterations in the human SEZ at one time point; however, it is of high value considering the pronounced interspecies differences in neurogenesis and migration between rodents and humans [3, 9-11, 34].

The role of IGF1 signalling during aging remains unclear in many aspects [35]. Our results support studies in mice showing that IGF1 is important for neuronal differentiation in the hippocampus [36] and neuronal migration to the olfactory bulb [37]. Thus, the function of IGF1 in adult neurogenesis may be conserved across species and neurogenic regions. IGF1 restoration rescues the age-related decline in hippocampal neurogenesis and cognitive impairments in rodents [31, 32, 38] and represents a putative therapeutic target for neurodegenerative and neurodevelopmental disorders [39, 40]. In contrast, downregulation of peripheral IGF1 signalling by genetic mutations delays aging and increases longevity [41-43]. IGF1R suppression in neural stem cells in the rodent SEZ prevents the age-related decrease in neurogenesis and olfactory deficits. This proliferation-promoting effect of reduced IGF1 signalling may prematurely deplete the neural stem cell pool; however, *in silico* modelling predicts that the number of stem cells is preserved until late adulthood [44] and accords with findings in the aged human SEZ [6, 7]. Long-lived Ames dwarf mice deficient in peripheral IGF1 show increased levels of hippocampal IGF1, suggesting that peripheral levels may negatively feedback on local IGF1 synthesis. Ames dwarf mice have increased neurogenesis and maintain normal cognitive function until advanced age [45]. These opposing reports highlight the need for further studies to discern the complex role of IGF1 signalling during aging in the mammalian brain.

The age-related decrease in IGF1 mRNA in the SEZ was not unexpected, although important to document in humans; however, it was unexpected that none of the other IGF family members declined with age. Since IGF1 was the only transcript to change significantly throughout adulthood, there may be an age-related alteration in control of gene expression specific to IGF1, such as methylation, histone modifications or loss of transcriptional activators. Age-related changes in local and peripheral neurogenesis-regulating factors may act in concert to decrease Ki67 and DCX expression in the SEZ across the human lifespan [5, 7, 13, 46]. Ki67 relates to proliferation of cell types other than neural stem cells such as astrocytes and microglia but displays a more robust expression in canonical and non-canonical neurogenic niches in the human brain [47]. Significant correlations of Ki67 with IGFBP2 and IGFBP4 as well as DCX with IGF1 and IGFBP2 suggest that IGF family members may work together to regulate adult neurogenesis in the human SEZ. IGFBP4 impairs proliferation and enhances neuronal differentiation of progenitor cells [25], whereas our results indicate that IGFBP4 may promote cell proliferation in conjunction with IGFBP2. We suggest that other mitogen signalling pathways in addition to IGF1 signalling may act independently or synergistically to stimulate cell proliferation as fibroblast growth factor receptor 1 expression also positively correlates with Ki67 mRNA [7]. IGF1 may act in concert with brain-derived neurotrophic factor and neuregulin signalling to promote neuronal differentiation as full-length tyrosine kinase receptor B and Erb-B2 receptor tyrosine kinase 4 expression positively correlate with DCX mRNA [7, 13]. In contrast, IGFBP2 may negatively regulate neuronal differentiation. It is unlikely that IGFBP2 acts in isolation as epidermal growth factor and truncated tyrosine kinase receptor B expression also negatively correlate with DCX mRNA [7, 13].

In summary, our results support that IGF family members impact the age-related decline in cell proliferation and neuronal differentiation markers in the human SEZ, though specific factors involved may depend on the stage of neurogenesis. We suggest that IGF family members only partially contribute to the complex local milieu available to regulate neurogenesis in the adult brain. This study is limited by the homogenate-based experimental approach and further cell-type specific analysis would shed light on whether proliferating cells and immature neurons maintain responsiveness to IGF1 during human aging. Gene expression studies also only provide clues as to whether protein levels may be altered and cannot ascertain if differences in brain protein levels may be of functional significance; however, several studies demonstrate that transcription changes in IGF family members significantly contribute to protein levels and to biological function [48-50]. We suggest that loss of local IGF1 function may impair neuronal differentiation and future work needs to establish if IGF1 restoration could rescue deficits in neurogenesis in the adult SEZ in the human brain.
